# TyG Index Change Is More Determinant for Forecasting Type 2 Diabetes Onset Than Weight Gain

**DOI:** 10.1097/MD.0000000000003646

**Published:** 2016-05-13

**Authors:** David Navarro-González, Laura Sánchez-Íñigo, Alejandro Fernández-Montero, Juan Pastrana-Delgado, Jose Alfredo Martinez

**Affiliations:** From the Garcia-Orcoyen Hospital (DN-G); Burlada Clinic (LS-Í), Navarra Health Service–Osasunbidea; Department of Occupational Medicine (AF-M), Preventive Medicine and Public Health; Department of Internal Medicine (JP-D), University of Navarra Clinic, Pamplona; IdiSNA—Health Research Institute of Navarra (JP-D, JAM); Food Science and Physiology (JAM), University of Navarra, Pamplona; and Centre of Biomedical Research in Pathophysiology of Obesity and Nutrition (CIBERObn) (JAM), Carlos III, Madrid, Spain.

## Abstract

The risk of type 2 diabetes associated with obesity appears to be influenced by other metabolic abnormalities, and there is controversy about the harmless condition of the metabolically healthy obese (MHO) state. The aim of this study is to assess the risk of diabetes and the impact of changes in weight and in triglyceride-glucose index (TyG index), according to the metabolic health and obesity states.

We analyzed prospective data of the Vascular Metabolic CUN cohort, a population-based study among a White European population (mean follow-up, 8.9 years). Incident diabetes was assessed in 1923 women and 3016 men with a mean age at baseline of 55.33 ± 13.68 and 53.78 ± 12.98 years old.

A Cox proportional-hazard analysis was conducted to estimate the hazard ratio (HR) of diabetes on metabolically healthy nonobese (MHNO), metabolically healthy obese, metabolically unhealthy nonobese (MUNO), and metabolically unhealthy obese (MUO). A continuous standardized variable (*z*-score) was derived to compute the HR for diabetes per 1-SD increment in the body mass index (BMI) and the TyG index.

MHO, MUNO, and MUO status were associated with the development of diabetes, HR of 2.26 (95% CI: 1.25–4.07), 3.04 (95% CI: 1.69–5.47), and 4.04 (95% CI: 2.14–7.63), respectively. MUNO individuals had 1.82 greater risk of diabetes compared to MHO subjects (95% CI: 1.04–3.22). The HRs for incident diabetes per 1-SD increment in BMI and TyG indexes were 1.23 (95% CI: 1.04–1.44) and 1.54 (95% CI: 1.40–1.68). The increase in BMI did not raise the risk of developing diabetes among metabolically unhealthy subjects, whereas increasing the TyG index significantly affect the risk in all metabolic health categories.

Metabolic health is more important determinant for diabetes onset than weight gain. The increase in weight does not raise the risk of developing diabetes among metabolically unhealthy subjects.

## INTRODUCTION

The link between generalized or central obesity and insulin resistance is currently accepted.^1^ However, some obese individuals appear to be at low risk of metabolic-related complications, whereas normal-weight individuals may not certainly be “healthy.” In this context, 2 terms have been used to identify the different statuses regarding metabolism and body size: metabolically healthy obese (MHO) and metabolically unhealthy nonobese (MUNO) individuals.^2,3^ Among obese individuals, those with preserved insulin sensitivity and lower inflammatory profile are classified as MHO.^4,5^ Subjects with higher levels of insulin resistance and adiposity, unfavorable lipid profile, and higher levels of proinflammatory cytokines are classified as MUNO.^6,7^ However, there is little agreement on the definition of metabolic health and obesity states leading to inconsistency in prevalence among the studies.^8,9^ In addition, there is controversy about the harmless condition of the MHO phenotype. Some studies have reported that MHO subjects had a greater risk of incident diabetes than nonobese subjects,^10–12^ whereas others have contradicted these results.^13–15^ Moreover, these are not stable conditions, and changes in body weight or metabolic health status might lead to different health consequences. Indeed, some studies have indicated that a proportion of those MHO at baseline might have been at the initial period of a metabolically unhealthy state.^16^

The triglyceride-glucose index (TyG index), the product of fasting plasma glucose and triglycerides, has been suggested as a marker of moderate insulin resistance,^17^ correlated with the M rates in the hyperinsulinaemic-euglycaemic clamp test ^18^ and with the homeostatic model assessment of insulin resistance (HOMA-IR).^19^ This measurement has also been suggested as a candidate marker for classifying the metabolic health status ^20^ and, recently, Lee et al ^21^ showed that changes in the TyG index over time altered the incidence and risk of diabetes.

Therefore, the aim of this study was to assess the incidence of type 2 diabetes according to the metabolic health and obesity status of 4939 free diabetes participants during 8.86 years of follow-up. We also studied the impact of the gain in body mass index (BMI) and in the TyG index on the risk of developing type 2 diabetes.

## METHODS

### Subjects

**FIGURE 1:**
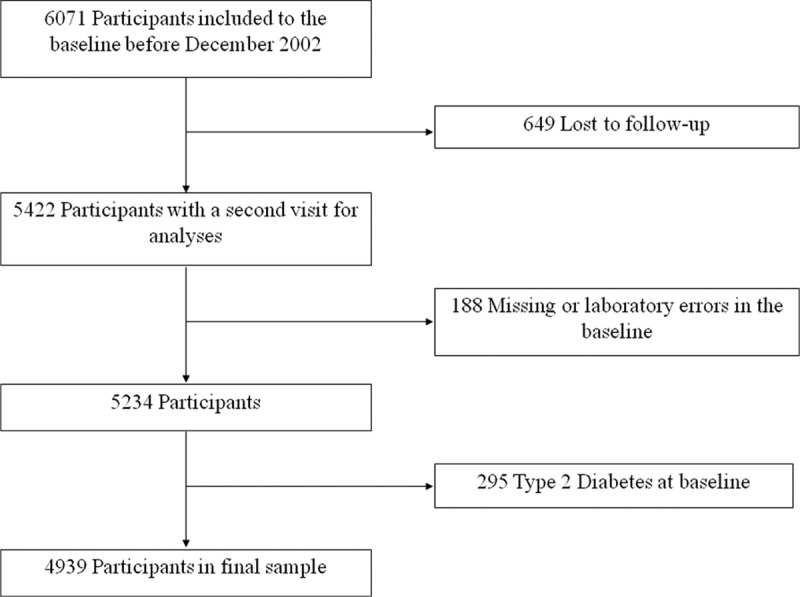
Flowchart of study participants drawn from the Vascular-Metabolic CUN clinical cohort between 1997 and 2012. CUN = University of Navarra Clinic.

The Vascular Metabolic CUN cohort (VMCUN cohort) is a population-based, epidemiological study designed to examine the incidence of cardiovascular and metabolic diseases including type 2 diabetes, hypertension, obesity, stroke, or coronary heart disease in a large White European population. The cohort has been described elsewhere.^22^ Briefly, exclusion criteria were age <18 or >90 years, history of type 1 diabetes or latent autoimmune diabetes in adults, cancer in the palliative phase, familial hypertriglyceridemia, extreme BMI (>45 kg/m^2^), or a hypercoagulable state. A total of 6071 people fulfilled the inclusion criteria. We excluded patients with prevalent diabetes, missing laboratory values, and those subjects lost to follow-up. Furthermore, since we wanted to study gain in BMI and TyG index, we restricted our analyses to those who had >1 follow-up examination. This left 4939 participants for the current analysis (Figure 1). The research was conducted according to the standards of the Declaration of Helsinki on medical research and was approved by the Ethics Committee of the University of Navarra (30/2015)**.**

## MEASUREMENTS

Data regarding medical history, health-related behaviors and serum biochemical measurements were retrieved at each patients’ visit. The median of follow-up was 10 years (mean 8.9 ± 4.3 years), with a median number of 3 visits per patient (range 2–8 visits) and a median time gap of 2 years between each clinical visit.

Health-related behaviors including cigarette smoking (none, former smoker, or current smoker), daily alcohol intake (yes/no), and lifestyle pattern (physically active/sedentary behavior) were obtained by physicians at the consultation. Before the measurement of blood pressure (BP), subjects waited for 5 minutes in a seated position. The BP on the indistinctly right or left arm was measured twice and the average value was recorded following World Health Organization criteria. Hypertension was defined on the basis of the World Health Organization-International Society of Hypertension Guidelines^23^ as ≥140 (systolic BP)/90 (diastolic BP) mm Hg or when the subjects reported use of antihypertensive medication. Cardiovascular disease was defined according to the International Classification of Diseases,^24^ Tenth Revision (ICD-10). The code list covers diseases from 3 groups: coronary heart disease, codes from I20 to I25; cerebrovascular disease, codes from I63 to I66; and peripheral arterial disease, codes I73.9 and I74. Anthropometric measurements and measurements of biochemical parameters including fasting plasma glucose (FPG), total cholesterol (TC), triglycerides (TG), HDL-cholesterol (HDL-C), and LDL-cholesterol (LDL-C) were obtained as it was described elsewhere.^22^ TG was measured using enzymatic colorimetric tests, and LDL-C was calculated using the Friedewald formula.^25^ The values of LDL-C were considered as missing in patients with TG levels >400 mg/dL. The TyG index was calculated as the ln[fasting TG (mg/dL) × FPG (mg/dL)/2].^18^ The TG/HDL-C ratio was calculated as TG divided by HDL-C (expressed in mg/dL).

### Definitions of Metabolic Health and Obesity States

Obesity phenotypes were defined according to the World Health Organization criteria: nonobese <30 kg/m^2^ and obese ≥30 kg/m^2^.^26^ The cut off TyG index levels to define a metabolically healthy state ^20^ were: TyG index <8.73 for women and <8.82 for men.

Metabolically healthy state was also defined using the Adult Treatment Panel III (ATP-III) components of the metabolic syndrome.^27^ The waist circumference (WC) was not used because of its collinearity with BMI. Participants who met three of the following criteria were considered metabolically healthy: (1) TG < 150 mg/dL; (2) HDL-cholesterol ≥40 mg/dL for men and ≥50 mg/dL for women; (3) blood pressure (BP) < 130/85 mm Hg; or (4) FPG <100 mg/dL. All individuals currently taking a pharmacological treatment for hypertension were assumed to have raised BP.

According to these criteria, study participants were categorized into 1 of 4 categories: (1) metabolically healthy nonobese (MHNO); (2) metabolically healthy obese (MHO); (3) metabolically unhealthy nonobese (MUNO); (4) metabolically unhealthy obese (MUO).

### Definition of Incident Diabetes

The diagnosis of type 2 diabetes was defined as the primary outcome of the study. We diagnosed diabetes according to the American Diabetes Association (ADA) ^28^: symptoms of diabetes plus random plasma glucose concentration ≥200 mg/dL (11.1 mmol/L), or FPG ≥126 mg/dL (7.0 mmol/L), or 2-h postload glucose ≥200 mg/dL (11.1 mmol/L) during an OGTT. From February 2010 onwards, we diagnosed diabetes according to the update ADA criteria published in 2010,^29^ which include the criteria of levels of HbA1c ≥6.5%. Each criterion, in the absence of unequivocal hyperglycemia, was achieved by repeated testing on a different day.

### Statistical Analysis

BMI and TyG index changes were calculated as the differences in weight and in the TyG index from each pair of consecutive visits (e.g., visit 2 minus visit 1, visit 3 minus visit 2, etc.). Continuous variables were expressed as the mean ± standard deviation (SD). Categorical variables were presented as percentages. Student's *t* test, 1-way ANOVA, or χ^2^ test were used to compare the baseline characteristics of study participants according to the metabolic health and obesity status. Age was the underlying time variable and exit-time was defined as date of diabetes for outcomes or date completing the last follow-up for survivors. We conducted a Cox proportional-hazard analysis to estimate the hazard ratio (HR) and their 95% confidence interval (CI) of type 2 diabetes according to the metabolic health and obesity status. The MHNO category was used as a reference. We fitted 3 models: a crude (univariate) model and 2 Cox regression multivariate-adjusted models: (a) controlling for age (continuous) and sex; (b) additionally adjusted for baseline BMI (continuous), cigarette smoking (never, current, and former smokers), daily alcohol intake (yes/no), lifestyle pattern (physically active/sedentary behavior), hypertension (yes/no), cardiovascular disease (yes/no), antiaggregation therapy (yes/no), LDL-C (continuous), HDL-C (continuous), and TG (continuous).

Secondary analyses were conducted to ascertain the impact of the gain in BMI and in the TyG index on the risk of developing diabetes. BMI and TyG index changes were recorded into quintiles. The category containing zero value was used as the reference group. Linear trend estimation model was used to fit the median of the quintiles as a continuous variable to estimate the trend of diabetes incidence across quintiles. We then derived a continuous standardized variable (*z*-score; mean = 0, SD = 1) to compute the HR for incident diabetes per 1-SD increment in BMI and TyG indexes. In addition, we performed stratified analyses in prespecified subgroups defined by age (<60 vs ≥60 years old), BMI categories (normal weight = 18.5–24.9 kg/m^2^, overweight = 25–29.9 kg/m^2^, and obesity = 30 kg/m^2^ or greater), sex (men vs women), and participants without hypertension and cardiovascular disease at baseline.

Interaction terms between the metabolic health and obesity status or BMI and TyG index gained and subgroups characteristics were tested using likelihood ratio comparing models with and without multiplicative interaction terms. The multiple imputation procedure was applied to impute the missing data of the variables cigarette smoking (16.6% missing values), daily alcohol intake (25.4% missing values), and lifestyle pattern (30.8% missing values). Twenty imputed datasets were created to reduce sampling variability from the imputation process. The variables included in the imputation procedure were: age, sex, BMI, cardiovascular disease, hypertension, TyG index, FPG, TG and the outcome, type 2 diabetes. A run length of 100 iterations was used between each data set. All the variables included were tested and present a normal distribution.

All statistical analyses were performed with STATA version 12 (Stata Corp., College Station, TX). All *P*-values are 2-tailed and statistical significance was set at the conventional cut-off of *P* < 0.05.

## RESULTS

### Baseline Characteristics

Data from 1923 women and 3016 men with a mean (±SD) age at baseline of 55.33 ± 13.68 and 53.78 ± 12.98 years were observed for an average of 9.06 and 8.73 years, respectively.

Baseline clinical and characteristics of the study according to metabolic health categories are shown in Table 1. Overall, 67% of patients were categorized as MHNO, 14% as MHO, 12% as MUNO, and 7% as MUO. Compared with metabolically healthy individuals, metabolic unhealthy subjects showed higher frequencies of antiaggregation therapy, cardiovascular disease, current smokers, and daily drinkers.

**TABLE 1:**
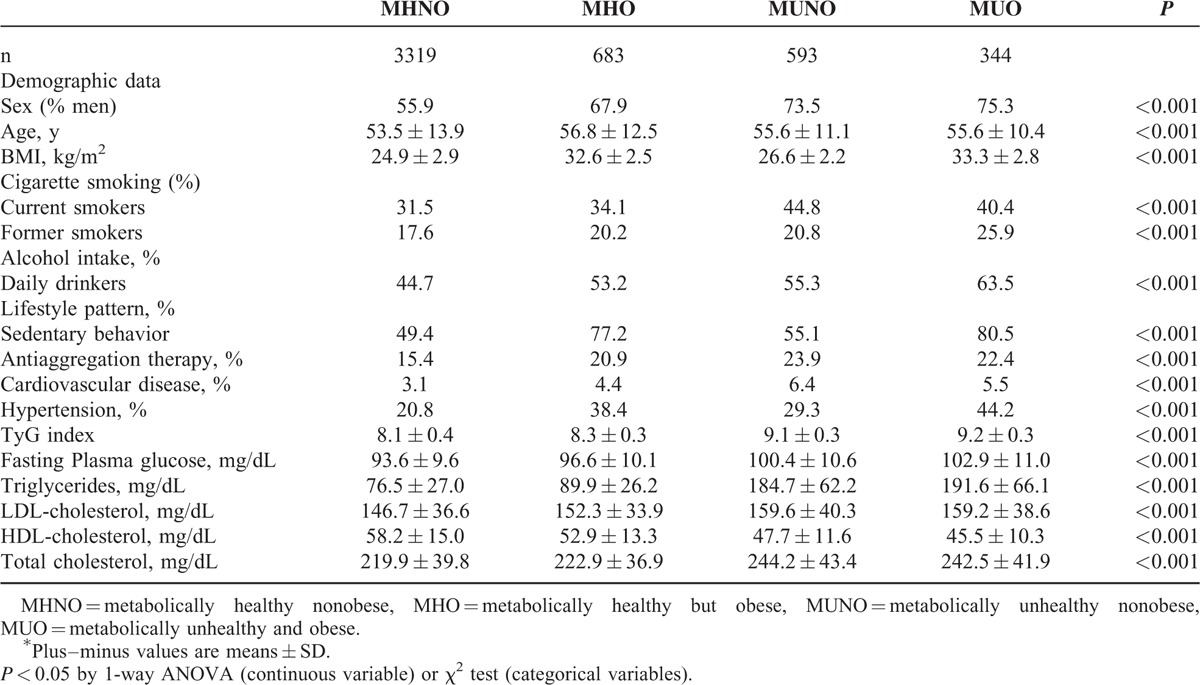
Characteristics of Study Participants According to Metabolic Health and Obesity States of 4939 Free-Diabetes Participants Drawn From the Vascular Metabolic CUN Clinical Cohort Between 1997 and 2012^∗^

### Risk of Incident Type 2 Diabetes According to the Metabolic Health Categories

There were 406 cases of incident type 2 diabetes during 44115.67 person-years of follow-up (overall incidence of 8.22 % or 9.21 cases/1000 person-years). Incidence was 5.67% for women and 9.84% for men. The incidence of type 2 diabetes in the category MUO was twice the incidence of MHO and MUNO (Table 2). MHO, MUNO, and MUO status were associated with the development of diabetes, compared with MHNO. The HR for the multivariate adjusted model were 2.26 (95% CI: 1.25–4.07), 3.04 (95% CI: 1.69–5.47), and 4.04 (95% CI: 2.14–7.63), respectively. The results did not change when the metabolic health phenotypes were analyzed with the ATP-III criteria. MUNO individuals had greater risk of incident diabetes compared to MHO subjects, HR 1.82, (95% CI: 1.04–3.22), in the multivariate adjusted model.

**TABLE 2:**
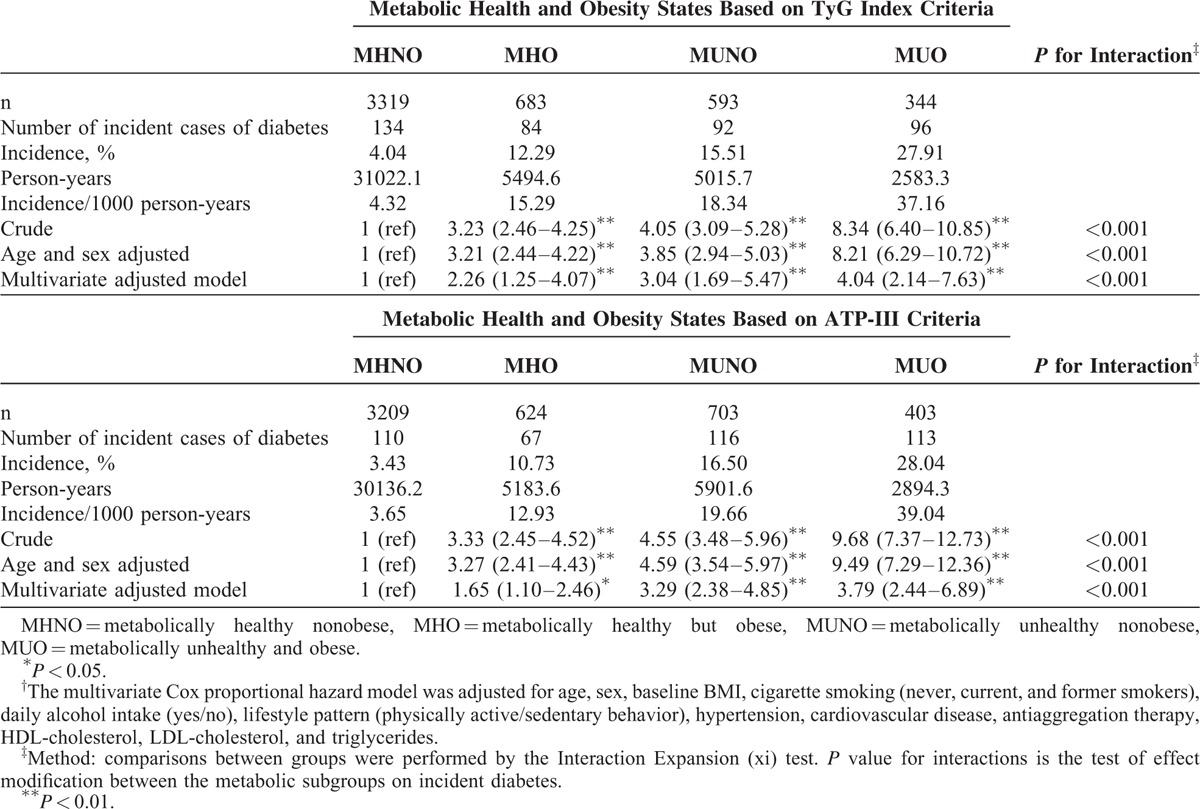
Risk of Incident Type 2 Diabetes According to the Metabolic Health and Obesity States of 4939 Free-Diabetes Participants Drawn From the Vascular Metabolic CUN Clinical Cohort Between 1997 and 2012^†^

### Risk of Incident Type 2 Diabetes and BMI and TyG Index Change

We found a significant increased risk of diabetes among the patients with increase of the BMI or TyG index (Table 3). The HR for incident diabetes for subjects in the fifth quintile of BMI change (>1.13 kg/m^2^) and TyG index change (>0.09 units) compared with stable category were 1.74 (95% CI: 1.15–2.62) and 4.78 (95% CI: 3.09–7.37), respectively. The risk of diabetes increased with increasing quintiles of BMI and TyG index change (*P* for trend <0.05).

**TABLE 3:**
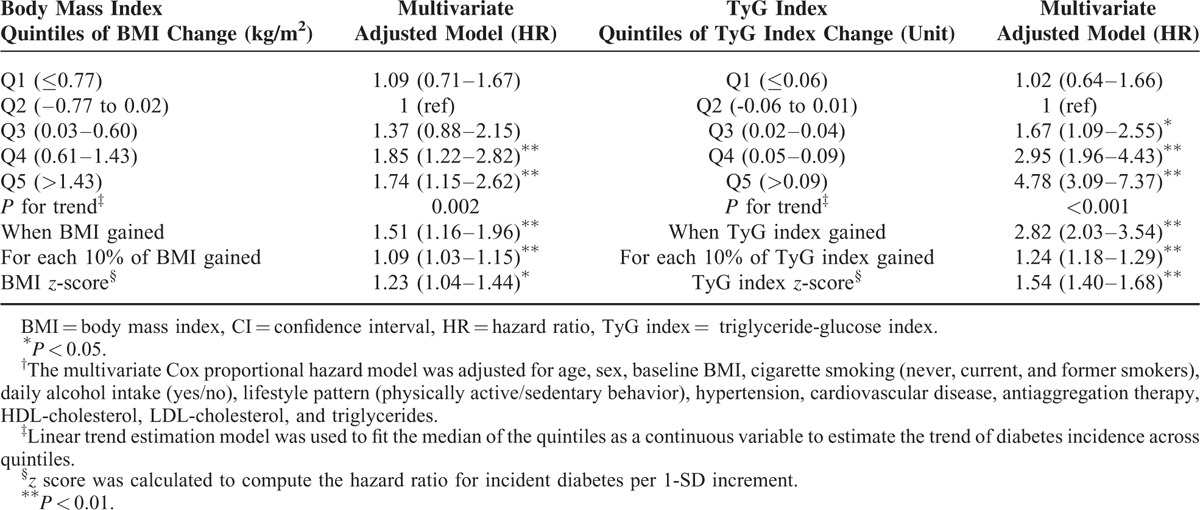
Risk of Incident Type 2 Diabetes According to Changes in the Body Mass Index or TyG Index During the Follow-Up of 4939 Free-Diabetes Participants Drawn From the Vascular Metabolic CUN Clinical Cohort Between 1997 and 2012^†^

**FIGURE 2:**
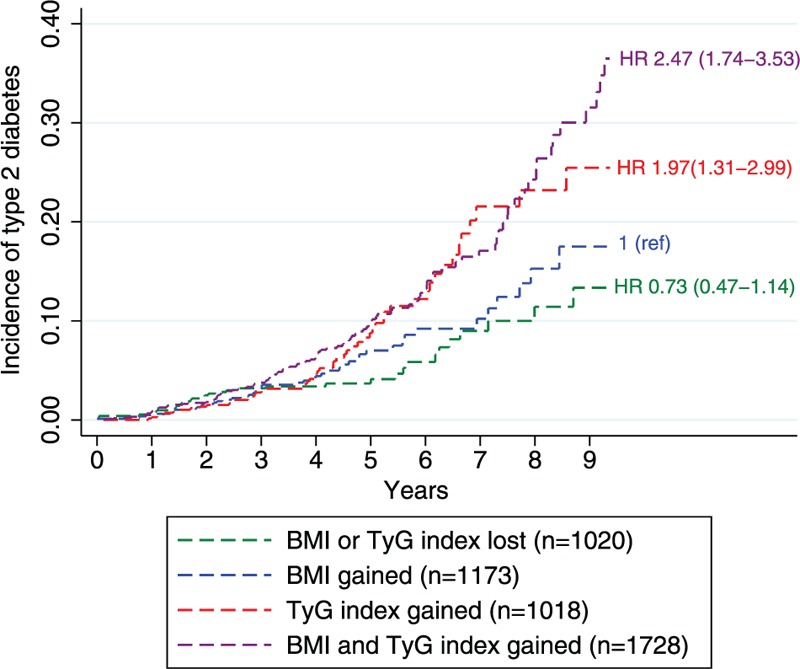
Cumulative incidence of diabetes by body mass index (BMI) gained, TyG index gained, both or none. Hazard ratio (HR) and their 95% confidence interval (CI) of developing diabetes were calculated after adjusting for age, sex, baseline BMI, cigarette smoking (never, current and former smokers), daily alcohol intake (yes/no), lifestyle pattern (physically active/sedentary behavior), hypertension, cardiovascular disease, antiaggregation therapy, HDL-cholesterol, LDL-cholesterol, and triglycerides. BMI = body mass index, CI = confidence interval, HR = hazard ratio, TyG index =  triglyceride-glucose index.

The cumulative incidence of diabetes raised proportionally if BMI increased, TyG index increased (HR: 1.97 [95% CI: 1.31–2.99]), and the increment of both parameters together (HR: 2.47 [95% CI: 1.74–3.53]) (Figure 2).

The main results of the present study were consistent for almost all the different scenarios that we included in the sensitivity analyses (Table 4). Metabolically unhealthy individuals who gained weight during the study did not show an increased risk of developing diabetes. In contrast, increasing the TyG index significantly affect the risk of future diabetes in all metabolic health categories and particularly in obese. The results were not different when the ATP-III criteria were used to define the metabolic health status. Only obese individuals who had gain in BMI showed an increased risk of diabetes, but the gain in the TyG index significantly increased the diabetes risk, independently of the BMI at baseline. The association of gain in BMI or TyG index with the risk of diabetes was similar across all the subgroups of the study population. Analysis without hypertension and cardiovascular disease at baseline were performed and the results of our study were not different.

**TABLE 4:**
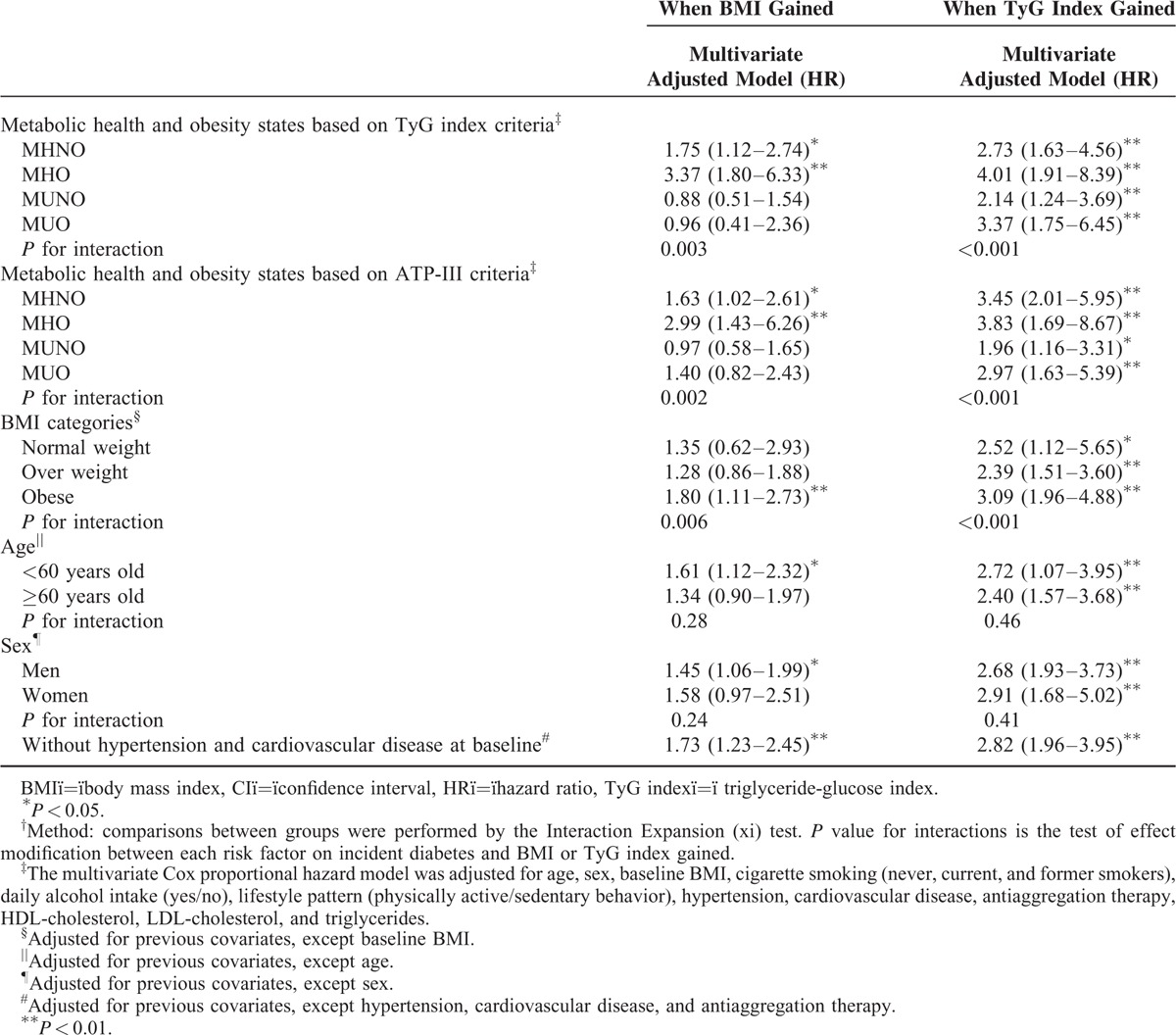
Stratified Analysis of Multivariate Hazard Ratios for Type 2 Diabetes as Affected by the Gain in the Body Mass Index or TyG Index^†^^,^

## DISCUSSION

In this large cohort study of White European population, subjects who were metabolically unhealthy (MUNO and MUO) showed an increased risk of diabetes incidence significantly greater than those subjects metabolically healthy, obese and nonobese. The results were similar when using the TyG index or the ATP-III criteria to define the metabolic health and obesity status of participants. The gain in BMI and in the TyG index was positively associated with the development of diabetes. Furthermore, the burden on diabetes incidence was considerably higher if the TyG index was raised. In addition, the increment in the TyG index was associated with type 2 diabetes in both metabolically healthy and unhealthy individuals, obese and nonobese. The gain in BMI was associated with diabetes only among metabolically healthy individuals.

Results from preceding studies proposed several mechanisms to explain the favorable metabolic profile of MHO subjects, such as the preserved insulin sensitivity,^30^ lower visceral and ectopic fat distribution,^5^ lower concentrations of tumor necrosis factor-α, interleukin-6 or adiponectin,^6,7^ as well as a favorable noninflammatory state.^4,31^ Visceral fat seems to be the starting point for insulin resistance, where lipolysis is enhanced and the flux of free fatty acids is altered. On the other hand, subcutaneous fat appears to protect against insulin resistance and obesity.^32^ However, it remains unclear whether obese individuals who are metabolically healthy have an increased risk of type 2 diabetes over time. Several studies have reported that MHO subjects had a greater risk of diabetes incidence than MHNO subjects,^10–12^ whereas others have reported opposite findings.^13–15^ In fact, some studies have suggested that MHO status is not a permanent condition but a transitory state.^16,33^ In this context, Appleton et al^15^ showed that, though MHO subjects had an increased risk of diabetes, it was mainly among those who progressed to a metabolically unhealthy state over a time frame follow-up.

The results of this study are in line with foregoing ones suggesting that MHO would not be a harmless condition, regarding the development of diabetes and compared to the MHNO state, but also highlights that MUNO individuals had greater risk of incidence in diabetes compared to MHO subjects. The combination of being metabolically unhealthy and obese heightens the risk of diabetes incidence. Thus, metabolic health status is likely to be more important determinant for the risk of diabetes than BMI.^13,34^

To our knowledge, few studies have examined the relationship between changes in metabolic health status over time and the risk of developing diabetes.^21,34,35^ Our study is apparently the first to compare the effect of weight gain and metabolic health change on future development of diabetes.

In line with others studies,^15,16,36,37^ weight gain was associated with an increased risk of diabetes in both MHO and MHNO subjects, but the effect on diabetes incidence was lower. The lack of association between weight gain and the risk of diabetes in metabolically unhealthy individuals might be explained in part by the greater absolute risk for developing diabetes in this group, such that any additional risk factor was unlikely to contribute to the overall risk. The underlying insulin resistance of these metabolically unhealthy individuals could be another reason involved. Our findings are supported by experimental human studies, which suggested that the development of hyperinsulinemia and insulin resistance occurs during the early stage of weight gain.^38^ In fact, increasing BMI does increase the hazard ratio for diabetes, but, importantly, the cumulative incidence rates remain quite low. In contrast, a rise in the TyG index resulted in higher rates of incident diabetes regardless of BMI. However, the gain in BMI does interact with the increment of TyG index to significantly much higher cumulative incident rates. Recently, Wei et al^37^ reported that gaining in BMI was associated with higher risk of diabetes in the younger compared with middle age groups. Our study supports this age effect modification on diabetes risk: the raise in BMI did not improve the risk of diabetes in ≥60 year-old subjects, compared with <60 years old. Weight gain at a younger age may lead to an increase in visceral adiposity and inflammation, compared with older age.^39^

Such as an important difference between metabolically healthy and unhealthy subjects was the higher prevalence for atherogenic dyslipidemia, determined by lower HDL-cholesterol and hypertriglyceridemia. This association between atherogenic lipoprotein abnormalities and the developing of diabetes was described before.^40^ In this context, our group has recently reported the effect of high triglycerides levels on the risk of diabetes, and the usefulness of the TyG index to early identify individuals at risk of diabetes.^41^ The TyG index has been proposed as a surrogate of IR, significantly correlated with the M rates in the hyperinsulinemic-euglycemic clamp test,^18^ with the HOMA-IR,^19^ as well as with fat distribution, subclinical atherosclerosis ^17^ and diabetes incidence.^42^ Lee et al ^21^ reported that changes in the TyG index over time altered the incidence and risk of diabetes. In this context, the predictive power of the TyG index to early identify diabetes onset was compared with FPG and triglycerides.^41^ When analyzing the areas under the curves, the *P* value obtained was statistically significant, indicating that the TyG index was a better predictor than FPG (*P* = 0.004) and triglycerides (*P* = 0.034), when the glucose level was under 100 mg/dL. The areas under the ROC curves (95% CI) were 0.75 (0.70–0.81) for the TyG index, 0.66 (0.60–0.72) for FPG, and 0.71 (0.65–0.77) for triglycerides, respectively.

Multiple definitions of metabolic health have been used to define the metabolic health status.^8,9^ We used the TyG index to define metabolically unhealthy individuals based on the results of previous studies suggesting that the TyG index is a useful candidate marker for classifying metabolic health status.^20^ Indeed, when we used the ATP-III definition of metabolic syndrome to define metabolic health, we did not found differences. Taking into account the results of this study, we state that the TyG index might be a component in the future definition of a metabolically healthy status and it might be used to easily classify individuals as metabolically unhealthy.

The principal strength of this study is the fact that the anthropometric values and the serum biochemical data were directly obtained or measured throughout the follow-up period by trained nurses and physicians in a clinical setting, whereas other studies used self-reported data, which could lead to errors and bias. These impartial measurements are crucial for studying metabolic-weight change over time. As our data were repeated measurements, we could establish that the weight and metabolic health change were gradual or uneven during the period of the study.

On the other hand, this study also has some limitations. We did not collect data of nutritional habits or energy intake. Although we have not adjusted for this possible confounding factor, we used other additional variables to adjust, such as BMI or cholesterol levels which are indirectly related to nutritional habits.

Because we could not assess insulin secretion, some metabolically healthy individuals might have isolated insulin resistance without the major common metabolic abnormalities. Thus, we cannot deny the possibility of misclassification of some participants. That might also have influenced overestimating the HR for diabetes among MHO individuals. However, studying the changes in BMI and TyG index over time, as well as repeated measures, contributed to overcome, at least partially, this possible bias. Also the lack of information on the use of lipid-lowering therapy or antidiabetic drugs may have influenced the results. However, if this bias explained the findings, the expected change would be towards the null, not away from the null.

In conclusion, metabolically unhealthy individuals exhibited a greater risk of diabetes than metabolically healthy obese individuals. The increase in the TyG index might be more important for developing diabetes than simply weight gain. These findings suggest the importance of the metabolic health status in the risk of diabetes, in contrast of the obesity assessed by BMI. Our observations might imply a different intervention strategy for diabetes prevention according to the different metabolic health and obesity states.
